# Optimisation of care among patients with diabetes mellitus and acute coronary syndrome through a specialised cardiodiabetes service—A registry study

**DOI:** 10.1111/dme.70030

**Published:** 2025-04-02

**Authors:** Muhammad Usman Shah, Alun Roebuck, Bala Srinivasan, Paul Edward Squires, Claire Elizabeth Hills, Maxime Inghels, Kelvin Lee

**Affiliations:** ^1^ Cardiorenal Group, Diabetes, Metabolism, & Inflammation, Joseph Bank Laboratories University of Lincoln Lincoln UK; ^2^ Lincoln Heart Centre United Lincolnshire Hospitals Lincoln UK; ^3^ Lincoln Institute for Rural and Coastal Health University of Lincoln Lincoln UK; ^4^ Department of Diabetes and Endocrinology United Lincolnshire Hospitals Lincoln UK

**Keywords:** acute coronary syndrome, cardiovascular outcomes, diabetes mellitus, treatment optimisation

## Abstract

**Aims:**

Diabetes mellitus remains a prevalent condition worldwide and a significant risk factor for atherosclerotic cardiovascular disease. Recent evidence suggests the use of glucose‐lowering therapies with cardiovascular benefit in optimising the cardiometabolic profile of patients with type 2 diabetes mellitus. However, uptake remains low. This study was carried out to assess the impact of a novel cardiodiabetes service for the management of patients with diabetes mellitus presenting with acute coronary syndromes.

**Methods:**

A retrospective, observational, registry‐based analysis was performed among patients presenting with an acute coronary syndrome and diabetes mellitus to a regional heart centre before and after the implementation of a cardiodiabetes service. Intergroup comparison was made for the proportion of patients having a valid glycated haemoglobin during admission, initiation of guideline‐recommended glucose and lipid‐lowering therapies.

**Results:**

At median follow‐up of 29.7 months, a valid HbA1c measurement at baseline was lower in the pre‐intervention compared to the post‐intervention group (556/711 [78.2%] vs. 302/362 [83.4%], *p* = 0.043) while more patients in the post‐intervention group were prescribed sodium‐glucose co‐transporter inhibitors (297/362 [82.0%] vs. 359/711 [50.5%]). All‐cause mortality (5.2 vs. 12.3 [events/100 patient‐years], relative ratio [RR] 0.42, 95% confidence interval [CI] 0.28–0.61, and *p* < 0.001), first events of acute kidney injury (AKI) (10.0 vs. 13.0, RR 0.77, CI 0.57–1.03, *p* = 0.090) and all events of AKI (16.6 vs. 22.1, RR 0.75, CI 0.60–0.94, *p* = 0.015) were significantly lower in the post‐intervention group.

**Conclusion:**

The introduction of a joint‐speciality cardiodiabetes service improved the care and survival of patients with acute coronary syndrome and diabetes mellitus.

**Whats new?**

- Recent evidence has proven certain glucose‐lowering therapies such as sodium‐glucose co‐transporter inhibitors and glucagon‐like peptide receptor agonists to have cardiovascular benefits in patients with type 2 diabetes mellitus.
- Despite recommendations by international guidelines for these medications to be used as first‐line therapies in these patients, their uptake remains low.
- A novel cardiodiabetes service improves screening and monitoring of patients with diabetes mellitus when presenting with acute coronary syndrome and allows early optimisation of medical therapy resulting in improved mortality and renal outcomes.

## 1 BACKGROUND

Diabetes mellitus (DM) remains a prevalent and growing public health issue worldwide.^1^ It is an important risk factor for atherosclerotic cardiovascular disease (ASCVD) and prevalent in up to 30% of patients with ASCVD.^2^ Individuals with DM are more likely to have advanced ASCVD with poorer outcomes,^1^ as well as other conditions including heart failure, cerebrovascular accidents (CVA) and peripheral arterial disease. Cardiovascular diseases account for up to 50% of deaths in patients with DM.^3^ Early DM screening and DM control monitoring in patients with ASCVD, particularly those experiencing acute coronary syndrome (ACS), allow for timely diagnosis, optimised management, and can lead to improved outcomes.^4^

Significant developments have taken place with regards to managing DM, particularly type 2 diabetes mellitus (T2DM), in individuals with ASCVD. Several trials have now established cardiovascular benefits associated with certain classes of glucose‐lowering medications such as sodium‐glucose co‐transporter‐2 inhibitors (SGLT2is) and glucagon‐like peptide‐1 receptor agonists (GLP1RAs) in patients with established ASCVD in those with T2DM. Whilst insulin therapy remains the mainstay treatment for type 1 diabetes mellitus (T1DM), the European Society of Cardiology recommends SGLT2is as a first‐line drug therapy for ASCVD patients diagnosed with T2DM.^5^ However, the number of prescriptions for such medicines remains low.^6^, ^7^

Timely screening for the diagnosis of DM as well as the early initiation of the appropriate therapies is key to improving cardiovascular outcomes, in particular among those experiencing an ACS. To improve the timeliness of diagnosis and treatment optimisation, a novel cardiodiabetes service (CDS) for the management of patients with DM presenting with ACS at the Lincolnshire Heart Centre was developed. This study was carried out to assess its impact on patient care and outcomes. More specifically, we aimed to assess the proportion of patients who had a valid glycated haemoglobin (HbA1c) during admission, initiation of guideline‐recommended glucose and lipid‐lowering therapies and incidence of cardiovascular outcomes including events of hospitalisation for heart failure, myocardial infarction, hospitalisation for unstable angina, cerebrovascular accidents, acute kidney injury and death before and after implementation of the service.

## 2 MATERIALS AND METHODS

### 2.1 The cardiodiabetes service

The CDS was developed as a sub‐service and an integral part of the Lincolnshire Heart Centre, United Lincolnshire Hospitals Trust (ULHT). The service was aimed for patients with known or newly diagnosed DM (type 1, type 2 and maturity‐onset diabetes mellitus (MODY)) presenting with an ACS (inclusive of ST elevation myocardial infarction [STEMI], non‐ST elevation myocardial infarction [NSTEMI] or troponin negative unstable angina)^8^, ^9^, ^10^ and admitted to ULHT hospitals (i.e. Lincoln County Hospital, Grantham District Hospital and Pilgrim Boston Hospital). Patients who were pre‐diabetic or non‐diabetic, or, had a primary diagnosis other than ACS such as type 2 myocardial infarction or arrhythmias, were not eligible for the service. The service was delivered by a multi‐professional team of cardiology and diabetes consultant physicians, physician fellows and advanced clinical practitioners. The detailed description of the setup of the Cardiodiabetes Programme, including the clinical standard operating procedures for the service and the development of the cardiodiabetic registry, is available in the Supporting Information (Appendix S1).

### 2.2 Pseudonymisation, selection and eligibility

Pseudonymised data from the registry database were then used for analysis in this study. Patients admitted between 1st July 2018 and 31st December 2023 were included for analysis, with final data lock for analysis performed on 31st December 2024. All patients eligible for the CDS, and hence incorporated in the database, were included in the study. Inclusion criteria comprised a diagnosis of ACS with either a new or previously established diagnosis of DM (T1DM, T2DM or MODY). Patients who had a terminal event during the qualifying admission were excluded from the analysis.

#### 2.2.1 Diagnosis of diabetes

In patients without a previous history of DM, the World Health Organisation criteria for DM of either random blood glucose (≥11.1 mmol/L [200 mg/dL]), fasting blood glucose (≥7.0 mmol/L [126 mg/dL]) or HbA1c (≥48 mmol/mol [6.5%]) was used.^11^ In the context of an admission with ACS, a single measurement of HbA1c in the diabetic range (≥48 mmol/mol [6.5%]) is sufficient to make a diagnosis of diabetes, with the ACS event as an acute vascular complication of DM.^5^

### 2.3 Intervention Groups

Depending on whether patients attended the CDS, they were classified either into pre‐intervention (i.e. pre‐CDS) or post‐intervention (i.e. CDS) group. All individuals admitted prior to phase 1, and therefore not been able to use the service, were included in the pre‐intervention group. Individuals admitted after the start of phase 2 were included in the post‐intervention group. In the period following phase 1 initiation but prior to phase 2 commencement, due to existing clinical service pressures which were further aggravated by the Coronavirus disease pandemic, only a proportion of the patient who were eligible were reviewed in the clinic. Therefore, between the two phases, only those patients who attended the cardiodiabetes clinic were allocated to the post‐intervention group whilst the others into the pre‐intervention group.

### 2.4 Definition of outcome events

Myocardial infarction (MI) was defined as the development of symptoms of myocardial ischaemia or echo or imaging features of ischaemia with detection of rise or fall of cardiac troponin levels with one reading above the 99th percentile and comprised both ST elevation and non‐ST elevation MI.^12^ Hospitalisation for unstable angina (HUA) was defined as an unplanned urgent or emergency admission for crescendo or resting angina without a significant rise in cardiac troponin to qualify for MI. HHF was considered if a patient presented with clinical or radiological features of decompensated heart failure or received increased diuretic therapy for relief of symptoms. CVA was defined as the acute development of focal or global neurological deficit, presumed of vascular origin, lasting more than 24 h, whilst symptoms lasting less than 24 h were classified as a transient ischaemic event (TIA).^13^ Acute kidney injury (AKI) was diagnosed on a biochemical basis with at least a 50% increase in creatinine from baseline and outside of the normal range and associated with a decrease in estimated glomerular filtration rate (eGFR). Intergroup analysis was performed for the first events of HHF, MI/HUA, CVA/TIA, AKI and all‐cause death.

### 2.5 Analysis

Continuous variables were recorded as median with interquartile ranges. Intergroup comparisons were made for key clinical parameters as discussed above. Categorical variables were recorded as numbers with percentages and compared via chi‐square and Fisher's exact tests for significance, with a *p* ≤ 0.05 considered as significant. Event rates over a 24‐month period following discharge were presented in patient years with intergroup significance assessed with Poisson regression and Wald test with survival analysis performed using the Kaplan–Meier method and graphs generated accordingly. Relative ratios, confidence intervals and *p* values were presented accordingly. Patients who were lost of follow‐up were censored on the last review undertaken at local general practitioner surgery or by a hospital clinician.

Microsoft Excel 2016 and R version 4.4.1 were used for data storage and analysis respectively.^14^ Missing data were recorded in tables along with respective variables where applicable.

## 3 RESULTS

### 3.1 Study population and baseline characteristics

A total of 4471 patients presented with ACS to the Lincolnshire Heart Centre, of which 1160 (25.9%) patients had DM. Of these, 87 patients did not survive the qualifying ACS admission, leaving 1073 patients who were successfully discharged for outpatient follow‐up and included in the cardiodiabetes registry. Patients were followed up for a median of 29.7 IQR [19.5–42.7] months (37.0 [26.0–48.2] vs. 22.8 [16.3–27.4] in the pre‐ and post‐intervention groups, respectively, *p* < 0.001). Three hundred and sixty‐two patients were in the post‐intervention group and 711 in the pre‐intervention group. Baseline characteristics at admission are shown in Table 1 and were similar across both groups for most parameters. A greater proportion of patients in the post‐intervention group had established renal replacement therapy (1.1% vs. 0.3%, *p* = 0.188), previous renal transplant (1.1% vs. 0.3%, *p* = 0.188) and coronary artery bypass grafting (7.7% vs. 5.2%, *p* = 0.100) at baseline. In contrast, the pre‐intervention group had a higher incidence of atrial fibrillation (16.1% vs. 11.0%, *p* = 0.025), overall CKD (24.3% vs. 30.0%, *p* = 0.005) and AKI (11.9% vs. 16.6%, *p* = 0.041). Almost a third of the patients presented with STEMI, with a similar distribution among both groups (*p* = 0.924).

**TABLE 1:** Baseline characteristics of patients that were seen in the cardiodiabetic service in comparison to those that were not.

Characteristic	Overall, *N* = 1073^a^	Pre‐intervention, *N* = 711^a^	Post‐intervention, *N* = 362^a^	*p*‐value^b^
Age at admission (years)	70.0 (60.8, 78.2)	70.4 (61.0, 78.5)	69.2 (60.0, 77.3)	0.209
Sex				0.217
Male	781.0/1073.0 (72.8%)	509.0/711.0 (71.6%)	272.0/362.0 (75.1%)	
Female	292.0/1073.0 (27.2%)	202.0/711.0 (28.4%)	90.0/362.0 (24.9%)	
Smoking				0.098
Never smoked	350.0/896.0 (39.1%)	249.0/604.0 (41.2%)	101.0/292.0 (34.6%)	
Ex‐smoker	348.0/896.0 (38.8%)	221.0/604.0 (36.6%)	127.0/292.0 (43.5%)	
Current smoker	198.0/896.0 (22.1%)	134.0/604.0 (22.2%)	64.0/292.0 (21.9%)	
Unknown	154	91	63	
Systolic blood pressure at admission (mmHg)	143.0 (126.0, 160.0)	142.0 (126.0, 161.0)	144.0 (125.0, 160.0)	0.673
Unknown	1	1	0	
Heart rate at admission (beats/min)	80.0 (67.0, 92.0)	79.0 (67.0, 91.0)	80.0 (68.0, 92.0)	>0.938
Unknown	1	1	0	
Glucose at admission (mmol/l)	10.8 (8.1, 15.1)	11.0 (8.1, 15.3)	10.5 (8.0, 14.6)	0.181
	27	18	9	
BMI (kg/m^2^)	29.5 (26.3, 33.8)	29.4 (26.1, 33.7)	29.8 (26.6, 33.9)	0.581
	13	5	8	
Left ventricular ejection fraction (%)				0.562
Good (≥55%)	391.0/1043.0 (37.5%)	260.0/688.0 (37.8%)	131.0/355.0 (36.9%)	
Mildly impaired (45–54%)	256.0/1043.0 (24.5%)	165.0/688.0 (24.0%)	91.0/355.0 (25.6%)	
Moderately impaired (36–44%)	205.0/1043.0 (19.7%)	130.0/688.0 (18.9%)	75.0/355.0 (21.1%)	
Severely impaired (≤35%)	191.0/1043.0 (18.3%)	133.0/688.0 (19.3%)	58.0/355.0 (16.3%)	
	25	19	6	
Length of stay (days)	5.0 (3.0, 9.0)	5.0 (3.0, 9.0)	4.1 (3.0, 9.0)	0.253
Revascularisation				0.676
Full	655.0/1052.0 (62.3%)	428.0/693.0 (61.8%)	227.0/359.0 (63.2%)	
None	224.0/1052.0 (21.3%)	146.0/693.0 (21.1%)	78.0/359.0 (21.7%)	
Partial	173.0/1052.0 (16.4%)	119.0/693.0 (17.2%)	54.0/359.0 (15.0%)	
	14	5	9	
Chronic kidney disease	301.0/1073.0 (28.1%)	213.0/711.0 (30.0%)	88.0/362.0 (24.3%)	0.052
RRT	6.0/1073.0 (0.6%)	2.0/711.0 (0.3%)	4.0/362.0 (1.1%)	0.188
Renal transplant	6.0/1073.0 (0.6%)	2.0/711.0 (0.3%)	4.0/362.0 (1.1%)	0.188
History heart failure	86.0/1073.0 (8.0%)	60.0/711.0 (8.4%)	26.0/362.0 (7.2%)	0.474
History of ASCVD	375.0/1073.0 (34.9%)	262.0/711.0 (36.8%)	113.0/362.0 (31.2%)	0.067
History of CABG	65.0/1073.0 (6.1%)	37.0/711.0 (5.2%)	28.0/362.0 (7.7%)	0.100
Atrial fibrillation	158.0/1073.0 (14.7%)	117.0/711.0 (16.5%)	41.0/362.0 (11.3%)	0.025
AKI at admission	161.0/1073.0 (15.0%)	118.0/711.0 (16.6%)	43.0/362.0 (11.9%)	0.041
Diagnosis of diabetes				0.351
MODY	3.0/1073.0 (0.3%)	2.0/711.0 (0.3%)	1.0/362.0 (0.3%)	
T1DM	47.0/1073.0 (4.4%)	27.0/711.0 (3.8%)	20.0/362.0 (5.5%)	
T2DM	1023.0/1073.0 (95.3%)	682.0/711.0 (95.9%)	341.0/362.0 (94.2%)	
Diagnosis of ACS at discharge				0.809
ST elevation myocardial infarction	327.0/1073.0 (30.5%)	216.0/711.0 (30.4%)	111.0/362.0 (30.7%)	
Non‐ST elevation myocardial infarction	718.0/1073.0 (66.9%)	478.0/711.0 (67.2%)	240.0/362.0 (66.3%)	
Unstable angina	28.0/1073.0 (2.6%)	17.0/711.0 (2.4%)	11.0/362.0 (3.0%)	
Hba1c (mmol/mol [%])	61 [9.7] (51 [8.3], 77 [12.1])	61 [9.7] (51 [8.2], 77 [12.1])	62 [9.8] (53 [8.6], 73 [11.5])	0.500
LDL (mmol/L)	1.9 (1.3, 2.8)	2.0 (1.3, 2.8)	1.9 (1.4, 2.8)	0.900
TG (mmol/L)	1.6 (1.6)	1.6 (1.6)	1.8 (1.3)	0.100
Total Cholesterol (mmol/L)	3.9 (3.2, 5.0)	3.9 (3.2, 5.0)	3.9 (3.2, 5.1)	>0.999
HDL (mmol/L)	1.1 (0.9, 1.3)	1.1 (0.9, 1.3)	1.0 (0.9, 1.2)	0.200
Total/HDL ratio	3.6 (2.9, 4.9)	3.6 (2.8, 4.9)	3.9 (2.9, 5.1)	0.078
GFR (mL/min)	63.0 (46.0, 78.0)	62.0 (46.0, 77.0)	68.5 (49.3, 80.0)	0.016
Follow‐up Length Months	29.7 (19.5, 42.7)	37.1 (26.0, 48.2)	22.9 (16.3, 27.4)	<0.001

Abbreviations: ACS, acute coronary syndrome; ASCVD, atherosclerotic cardiovascular disease; BMI, body mass index; CABG, coronary artery bypass grafts; GFR, glomerular filtration rate; HbA1c, glycated haemoglobin; HDL, high‐density lipoprotein; LDL, low‐density lipoprotein; MODY, maturity‐onset diabetes of the young; RRT, renal replacement therapy; T1DM, type 1 diabetes mellitus; T2DM, type 2 diabetes mellitus; TG, triglycerides.

^a^ Median (25%,75%); *n*/*N* (%) mean (SD); *n*/*N* (%).

^b^ Wilcoxon rank sum test; Pearson's chi‐squared test; Fisher's exact test.

### 3.2 Diabetes screening and medicines optimisation

**FIGURE 1:**
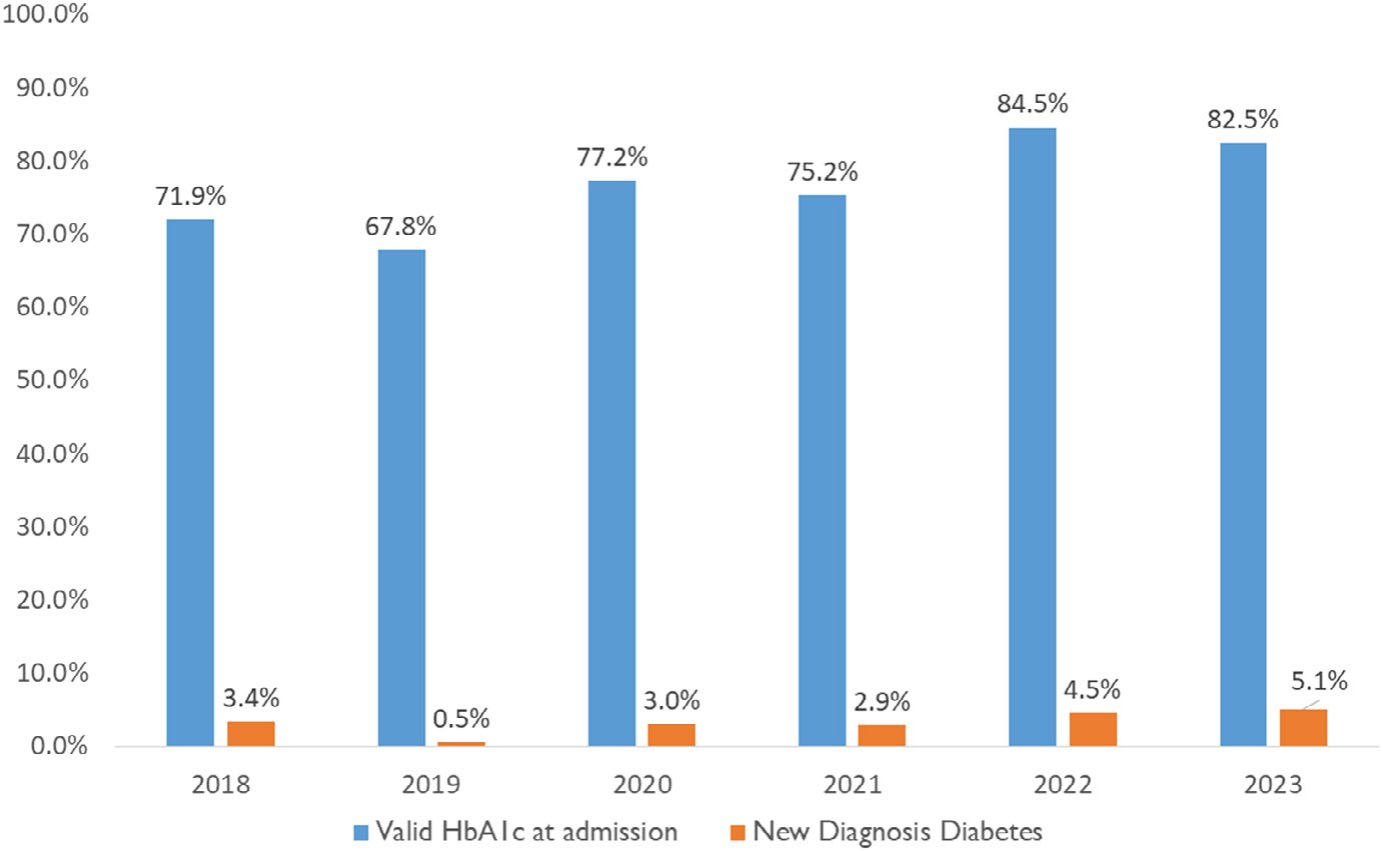
Screening and new diagnosis of diabetes mellitus via HbA1c in patients admitted with an ACS. Cardiodiabetic clinic started in summer 2021 and active in‐reach service commenced in autumn 2022.

**FIGURE 2:**
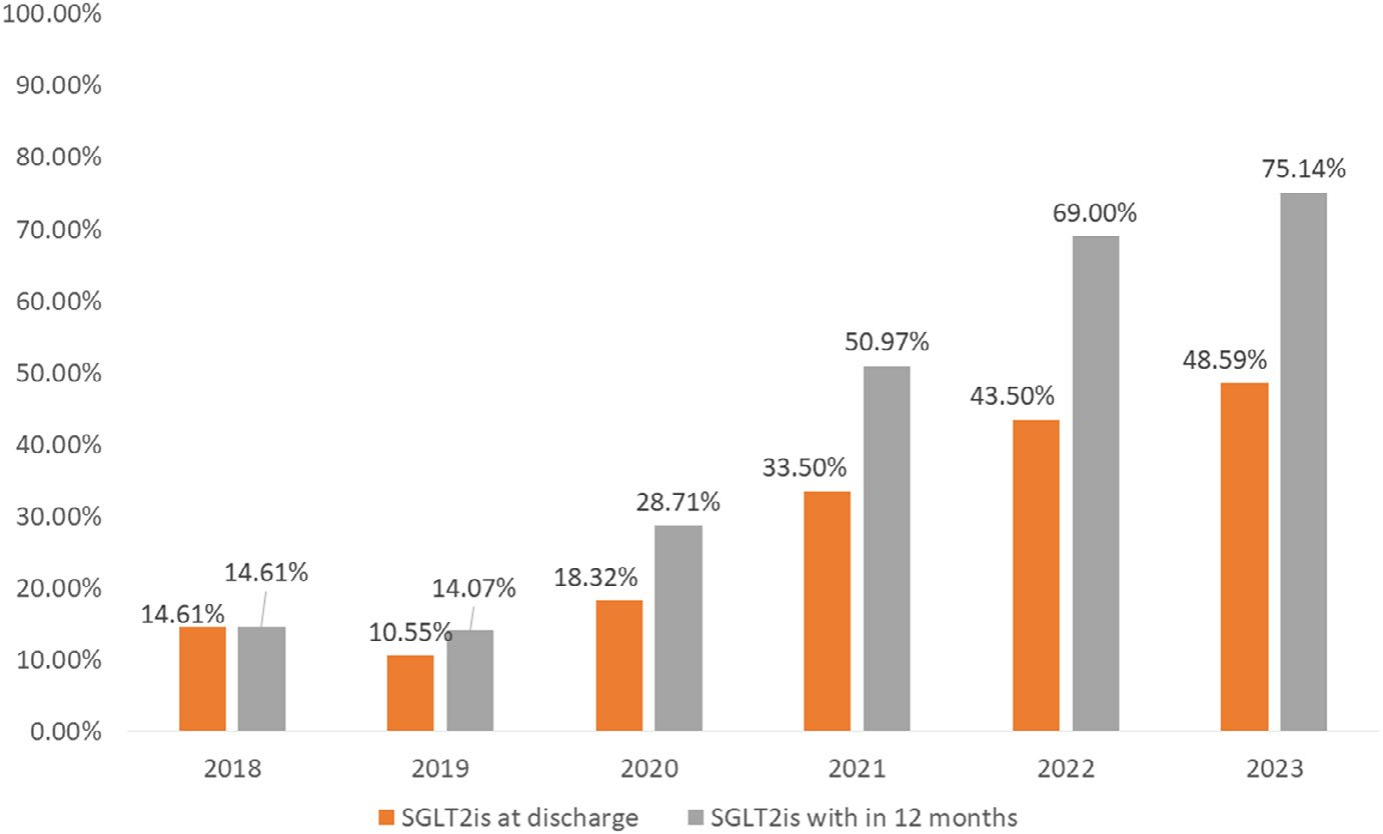
Prescription of SGLT2is among eligible patients with T2DM and ACS on a yearly: Cardiodiabetic programme established in 2021. Cardiodiabetic clinic started in summer 2021 and active in‐reach service commenced in autumn 2022.

The proportion of patients who had an HbA1c measurement at admission or within 3 months prior to admission initially reduced from 72/89 (80.9%) in 2018 to 149/199 (74.9%) by 2019 and then, during the intervention period, increased to 177/200 (88.5%) by 2022 (Figure 1). Overall, valid HbA1c measurement coverage at baseline was lower in the pre‐intervention compared to the post‐intervention group (556/711 [78.2%] vs. 302/362 [83.4%], *p* = 0.043). A greater proportion of patients had a new diagnosis of T2DM in the post‐intervention group in comparison to the pre‐intervention group (21/362 [5.8%] vs. 13/711 [1.8%], *p* < 0.001). More patients in the post‐intervention group were prescribed SGLT2is (297/362 [82.0%] vs. 359/711 [50.5%], *p* < 0.001) [Table 2]. The proportion of patients who were given a prescription of SGLT2is at discharge increased from 13/89 (14.6%) patients in 2018 to 86/177 (48.6%) patients by 2023 (Figure 2). A similar trend for the SGLT2is prescription within 12 months was seen.

**TABLE 2:** Medication prescriptions for the patients seen in the cardiodiabetes service and the patients seen before the service was established.

Medication prescribed	Overall, *N* = 1073^a^	Pre‐intervention, *N* = 711^a^	Post‐intervention, *N* = 362^a^	*p*‐value^b^
Aspirin	962.0/1073.0 (89.7%)	612.0/711.0 (86.1%)	350.0/362.0 (96.7%)	<0.001
Non‐aspirin antiplatelet	1002.0/1073.0 (93.4%)	659.0/711.0 (92.7%)	343.0/362.0 (94.8%)	0.198
Beta‐blockers	978.0/1073.0 (91.1%)	639.0/711.0 (89.9%)	339.0/362.0 (93.6%)	0.040
ACEi/ARBs	941.0/1072.0 (87.8%)	622.0/710.0 (87.6%)	319.0/362.0 (88.1%)	0.807
Entresto	105.0/1073.0 (9.8%)	70.0/711.0 (9.8%)	35.0/362.0 (9.7%)	0.927
Aldosterone antagonists	297.0/1073.0 (27.7%)	190.0/711.0 (26.7%)	107.0/362.0 (29.6%)	0.326
Glucose‐lowering agents				
SGLT2i Prescribed overall				<0.001
Yes	656.0/1073.0 (61.1%)	359.0/711.0 (50.5%)	297.0/362.0 (82.0%)	
Contraindicated	103.0/1073.0 (9.6%)	68.0/711.0 (9.6%)	35.0/362.0 (9.7%)	
SGLT2i				<0.001
Before admission	159.0/1073.0 (14.8%)	73.0/711.0 (10.3%)	86.0/362.0 (23.8%)	
At admission	154.0/1073.0 (14.4%)	79.0/711.0 (11.1%)	75.0/362.0 (20.7%)	
Post‐discharge CD	98.0/1073.0 (9.1%)	0.0/711.0 (0.0%)	98.0/362.0 (27.1%)	
Post‐discharge other	245.0/1073.0 (22.8%)	207.0/711.0 (29.1%)	38.0/362.0 (10.5%)	
Sulfonyl urea	232.0/1073.0 (21.6%)	171.0/711.0 (24.1%)	61.0/362.0 (16.9%)	0.007
DPP4i	294.0/1073.0 (27.4%)	200.0/711.0 (28.1%)	94.0/362.0 (26.0%)	0.453
Thiazolidinediones	5.0/1073.0 (0.5%)	5.0/711.0 (0.7%)	0.0/362.0 (0.0%)	0.174
Metformin	682.0/1073.0 (63.6%)	451.0/711.0 (63.4%)	231.0/362.0 (63.8%)	0.903
GLP1RA	156.0/1073.0 (14.5%)	98.0/711.0 (13.8%)	58.0/362.0 (16.0%)	0.325
Insulin	386.0/1073.0 (36.0%)	257.0/711.0 (36.1%)	129.0/362.0 (35.6%)	0.869
Lipid‐lowering therapies				
Statin	1022.0/1060.0 (96.4%)	677.0/708.0 (95.6%)	345.0/352.0 (98.0%)	0.059
Ezetimibe	279.0/1070.0 (26.1%)	124.0/708.0 (17.5%)	155.0/362.0 (42.8%)	<0.001
Icosapent ethyl	30.0/1073.0 (2.8%)	5.0/711.0 (0.7%)	25.0/362.0 (6.9%)	<0.001
Bempedoic acid	28.0/1073.0 (2.6%)	13.0/711.0 (1.8%)	15.0/362.0 (4.1%)	0.051
Fibrates	17.0/1073.0 (1.6%)	14.0/711.0 (2.0%)	3.0/362.0 (0.8%)	0.157
Inclisiran/PCSK9i	12.0/1073.0 (1.1%)	8.0/711.0 (1.1%)	4.0/362.0 (1.1%)	1.000

Abbreviations: ACE*i*, angiotensin‐converting enzyme inhibitors; ARBs, angiotensin receptor blockers; DPP4i, dipeptidyl peptidase inhibitors; GLP1RA, glucagon‐like peptide‐1 receptor agonist; PCSK9i, proprotein convertase subtilisin/kexin type 9; SGLT2i, sodium‐glucose co‐transporter 2 inhibitor.

^a^ Mean (SD); *n*/*N* (%).

^b^ Wilcoxon rank sum test; Pearson's chi‐squared test; Fisher's exact test.

The prescriptions of other glucose‐lowering medications including metformin, insulin, GLP1RAs and dipeptidyl peptidase‐4 (DPP‐4) inhibitors remained similar while a reduction was seen for prescriptions of sulfonylureas and thiazolidinediones. SGLTI2is became the most frequently prescribed medications for diabetic patients with ACS by 2021 despite being primarily prescribed to those with T2DM, in line with the introduction of the service (Figure S1). The baseline median HbA1c was similar between the two groups (60.7 [50.8, 77.3] vs. 61.5 [53.0, 73.3], *p* = 0.500) with a sustained reduction in HbA1c in the subsequent months in the post‐intervention group compared to the pre‐intervention group. (Figure S2).

### 3.3 Trends in eGFR

Baseline eGFR was higher in the post‐intervention group (68.5 mL/min [49.3, 80.0], *p* = 0.016) compared to the pre‐intervention group (62.0 mL/min [46.0, 77.0]) (Table 1). Following discharge, there was a reduction in eGFR noted in both groups, with subsequent stabilisation/improvement in the first 3 months. However, a gradual decrease in eGFR was noted in the pre‐intervention group for the rest of the duration of follow‐up over the 18 months compared to the post‐intervention group (Figure S2).

### 3.4 Adjuvant lipid therapy prescriptions and trends in lipid profile

Adjuvant lipid‐lowering therapies, in addition to statin therapy, were more frequently prescribed among patients in the post‐intervention group with a significantly higher proportion of patients receiving ezetimibe (42.8% vs. 17.5%, *p* < 0.001), icosapent ethyl (6.9% vs. 0.7%, *p* < 0.001) and bempedoic acid (4.1% vs. 1.8%, *p* = 0.024) (Table 2). Similar median measurements of total cholesterol, low‐density lipoproteins, high‐density lipoproteins and triglycerides (Table 1) were observed at baseline. Initial decreases in levels were noted, similar to that noted with HbA1c, for both groups following which the levels stabilised, and no significant differences were seen between the groups over 18 months (Figure S2).

### 3.5 Cardiovascular or renal event, all‐cause mortality and hospitalisation outcomes

Individuals were followed up for a total of 2784 patient‐years, with 676 patient‐years in the post‐intervention group (362 patients with median 22.9‐month follow‐up) vs. 2108 patient‐years in the pre‐intervention group (711 patients with median 37.1‐month follow‐up).

**FIGURE 3:**
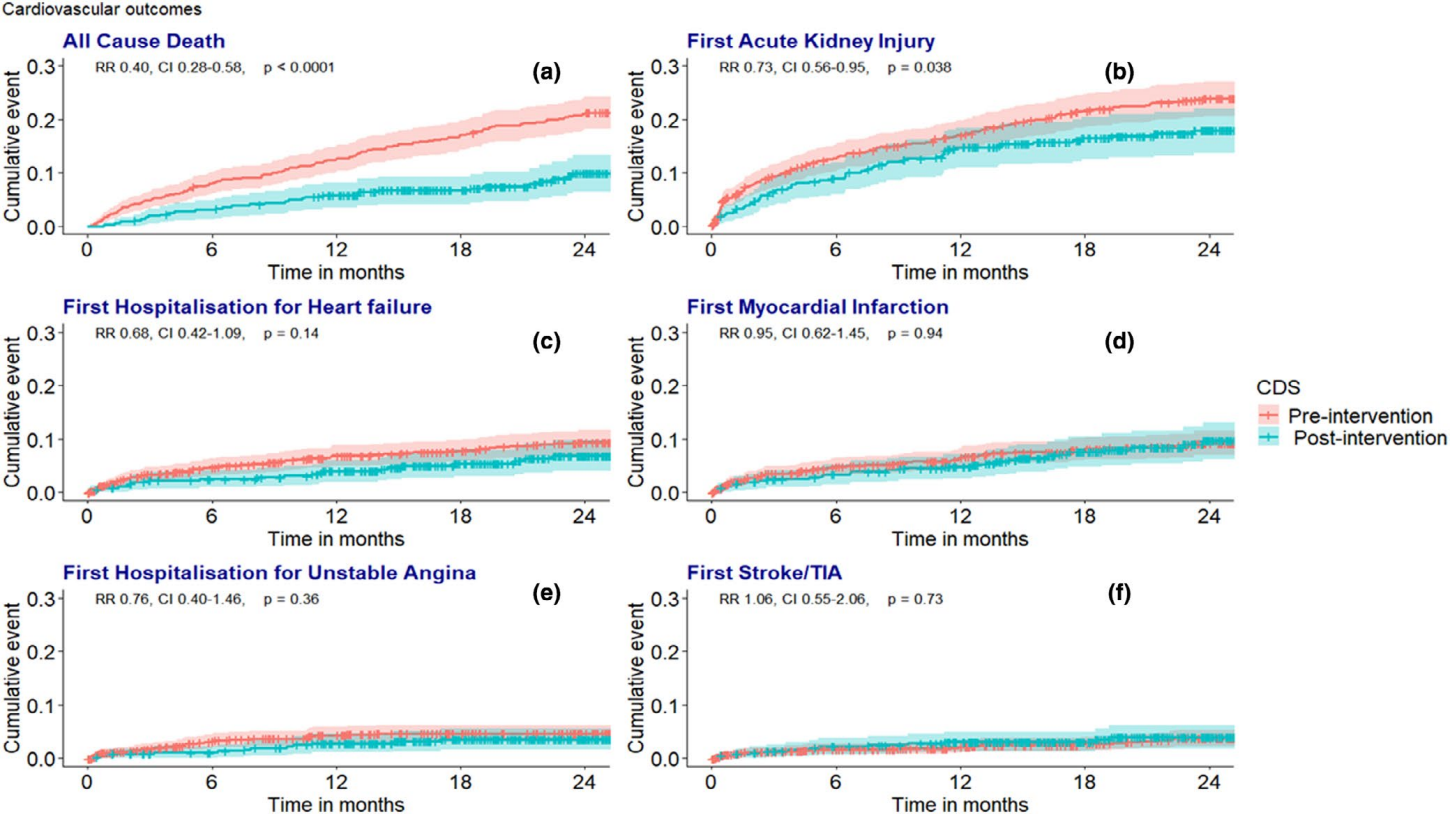
Kaplan–Meier graphs of first events of all‐cause death (a), acute kidney injury (b), hospitalisation for heart failure (c), myocardial infarction (d), hospitalisation for unstable angina (e) and stroke/transient ischaemic attack (TIA) (f).

At 24 months of follow‐up from discharge, 184 deaths occurred (31/362 vs. 153/711 in the post‐ and pre‐intervention group, respectively). All‐cause mortality was significantly lower in the post‐intervention group from the total study population (5.2 vs. 12.3 normalised to 100 patient‐years, relative ratio [RR] 0.42, 95% confidence interval [CI] 0.28–0.61, and *p* < 0.001, Table 3), with a similar trend noted for the first event of AKI (10.0 vs. 13.0 events per 100 patient‐years, RR 0.77, CI 0.57–1.03, *p* = 0.090, Table 3) and all events of AKI (16.6 vs. 22.1 events per 100 patient‐years, RR 0.75, CI 0.60–0.94, *p* = 0.015, Table 3). The cumulative Kaplan–Meier graphs demonstrate a significantly lower all‐cause death (RR 0.40, confidence interval 0.28–0.58, *p* < 0.001) and first AKI (RR 0.73, CI 0.56–0.95, *p* = 0.038) as shown in Figure 3. The incidence of HHF, MI/HUA and CVA/TIA remained similar between groups with no significant differences (*p* > 0.1, Table 3, and Figure 3).

**TABLE 3:** Incidence in patient years of first and all events of major outcomes between pre‐ and post‐intervention groups at 24 months of follow‐up from discharge.

Characteristic	Pre‐intervention^a^ (*N* = 711, follow‐up years = 1246)	Post‐intervention^a^ (*N* = 362, follow‐up years = 596)	RR, [95% CI], *p*‐value^b^
First HHF	4.9 (61)	3.5 (21)	0.72, [0.43–1.16], 0.193
Total HHF	6.7 (84)	5.9 (35)	0.87, [0.58–1.28], 0.491
First MI/HUA	6.4 (80)	6.0 (36)	0.94, [0.63–1.38], 0.759
Total MI/HUA	7.9 (98)	7.0 (42)	0.90, [0.62–1.28], 0.550
First CVA/TIA	1.9 (24)	2.2 (13)	1.13, [0.56–2.19], 0.719
Total CVA/TIA	2.0 (25)	2.3 (14)	1.17, [0.59–2.22], 0.638
First AKI	13.0 (162)	10.0 (60)	0.77, [0.57–1.03], 0.090
Total AKI	22.1 (275)	16.6 (99)	0.75, [0.60–0.94], 0.015
All‐cause death	12.3 (153)	5.2 (31)	0.42, [0.28, 0.61], <0.001
All‐cause events with hospitalisation	64.0 (797)	47.1 (281)	0.74, [0.64, 0.84], <0.001

Abbreviations: AKI, acute kidney injury; CI, confidence interval; CVA, cardiovascular accident (Stroke); HHF, hospitalisation for heart failure; HUA, hospitalisation for unstable angina; MI, myocardial infarction; RR, rate ratio; TIA, transient ischaemic attack.

^a^ Events per 100 patient‐years (total number of events).

^b^ Wald test.

Overall, 1078 all‐cause events requiring hospitalisations occurred within the 24 months from discharge, with 281 and 797 events in the post and pre‐intervention groups, respectively, and a significantly lower total event rate in the post‐intervention group (47.1 vs. 64.0 events per 100 patient‐years, RR 0.74, CI 0.64–0.84, *p* < 0.001) (Table 3).

### 3.6 Diabetes adverse event outcomes of interest

A total of 222 events occurred in the 24 months of follow‐up with no significant differences in total events between the groups, with 60 (10.1 events per 100 patient‐years) in the post‐intervention group and 162 (13.0 events per 100 patient‐years, RR 0.77, CI 0.57–1.03, *p* = 0.090) in the pre‐intervention group. Of the individual diabetic adverse events of interest, hypovolemia occurred less frequently in the post‐intervention group in comparison to the pre‐intervention one (1.2 vs. 2.7 events per 100 patient‐years, RR 0.43, CI 0.17–0.91, *p* = 0.042) and AKI which has been mentioned above. There were no other significant differences in individual diabetic adverse events of interest noted between the groups and only 2 patients developed necrotising fasciitis, both in the pre‐intervention group. The frequency of diabetic complications in pre‐intervention vs. post‐intervention groups is shown in Table 4.

**TABLE 4:** All diabetic complications as events per 100 patient‐years requiring hospitalisation or urgent treatment within 24 months of discharge.

Diabetic complication	Pre‐intervention^a^ (*N* = 711, follow‐up years = 1246)	Post‐intervention^a^ (*N* = 362, follow‐up years = 596)	RR, [95% CI], *p*‐value^b^
UTI	3.2 (40)	2.5 (15)	0.78, [0.42–1.39], 0.421
Hypoglycaemia	0.8 (10)	0.7 (4)	0.84, [0.23–2.50], 0.762
Hyperglycaemia	1.4 (17)	0.8 (5)	0.61, [0.20–1.55], 0.339
Hypovolemia	2.7 (34)	1.2 (7)	0.43, [0.17–0.91], 0.042
Amputation	0.8 (10)	1.2 (7)	1.46, [0.53–3.81], 0.440
Osteomyelitis	1.5 (19)	1.2 (7)	0.77, [0.30–1.75], 0.554
Diabetic foot necrosis	0.8 (10)	1.0 (6)	1.25, [0.43–3.38], 0.661
Infected ulcer	1.0 (12)	0.7 (4)	0.70, [0.19–2.00], 0.531
DKA	0.8 (10)	0.3 (2)	0.42, [0.06–1.59], 0.260
Abscess	0.2 (3)	0.3 (2)	1.39, [0.18–8.41], 0.717
Cellulitis	0.3 (4)	0.7 (4)	2.09, [0.49–8.84], 0.297
Fracture	0.2 (3)	0.2 (1)	0.70, [0.03–5.44], 0.754
All diabetic complications	13.0 (162)	10.1 (60)	0.77, [0.57–1.03], 0.090

Abbreviations: CI, confidence interval; RR, rate ratio; UTI, urinary tract infection.

^a^ Events per 100 patient‐years (Total number of events).

^b^ Wald test.

## 4 DISCUSSION

In this study, we reviewed the impact of a joint cardiology and diabetes speciality multi‐professional cardiodiabetes service to screen for, manage and optimise all patients with DM admitted with ACS at a regional heart centre. The new service led a more comprehensive screening and detection of diabetes and an up to date assessment of diabetes control (and of suboptimal control) at the time of admission leading to an earlier initiation of recommended diabetic medications particularly those of proven cardiovascular benefit. Prescribing behavioural changes were also observed with adjuvant lipid‐lowering therapies which were also more frequently prescribed in the post‐intervention group. A significant reduction in death was seen after the implementation of the service. Importantly, the early optimisation and use of diabetic medications with proven cardiovascular benefits did not lead to an increase in adverse diabetic complications. In contrast, the multidisciplinary management and optimisation of the cardiodiabetic patient led to a significant reduction in some adverse events such as hypovolemia and acute kidney injury post‐discharge.

A significant reduction in the incidence of all‐cause death and AKI events was observed after the implementation of the CDS. Whilst the CDS comprised a multifaceted approach towards early optimisation, one key intervention was the more frequent and earlier prescriptions of SGLT2is in the post‐intervention group. Similar results were seen with the EMPA‐REG OUTCOME (Empagliflozin Reduction in Glucose) trial showing a significant reduction in all‐cause deaths (5.7% vs. 8.3%, 32% RRR) among patients initiating Empagliflozin after the first ACS event, along with a reduction in kidney outcomes associated with SGLT2is in patients with DM.^15^, ^16^, ^17^ However, our analysis included patients with T1DM as well who are currently not licensed and therefore contraindicated to SGLT2i therapy and did not receive it in our cohort. Similarly, a greater proportion of patients in the post‐intervention group received adjuvant lipid‐lowering therapy including ezetimibe and icosapent ethyl which may have led to improved outcomes. The IMPROVE‐IT (Improved Reduction of Outcomes: Vytorin Efficacy International Trial) showed a significant reduction in major cardiovascular outcomes with ezetimibe when given in addition to a statin following an MI (hazard ratio 0.85; 95% confidence interval, 0.78–0.94; *p* < 0.001)^18^ with the REDUCE‐IT (Reduction of Cardiovascular Events with Icosapent Ethyl—Intervention Trial) trial showing similar results with Icosapent ethyl in those individuals with elevated fasting triglycerides (hazard ratio, 0.75; 95% confidence interval 0.68 to 0.83; *p* < 0.001).^19^ Whilst the combined early initiation of such indicated therapies may explain some of the benefit seen with the CDS intervention, we saw a greater reduction in death among our patients which may be further attributed to the holistic approach of optimising overall patient risk factors including lipid profile, in addition to improved patient screening and monitoring. Similarly, whilst the post‐intervention group had a lower incidence of AKI at admission (11.9% vs. 16.6%, *p* = 0.041), a higher proportion of patients with advanced kidney disease at baseline was noted in this group, including individuals with previous kidney transplant (1.1% vs. 0.3%, *p* = 0.2) and ongoing renal replacement therapy (1.1% vs. 0.3%, *p* = 0.2). Therefore, the post‐intervention group represented a relatively higher risk group with regards to renal outcomes and the benefit seen above in terms of reduction in such outcomes to be a possible beneficial impact from service implementation. Moreover, our data represents real‐world evidence where patients may be higher risk of further events and therefore, greater benefit from early initiation of indicated therapies.

Implementation of the CDS has led to greater screening and monitoring of patients with DM allowing early medication optimisation within the CDS Team as well as awareness amongst wider specialties such as acute medicine. Whilst random blood glucose monitoring may be frequently performed in patients with MI, screening for diabetes with HbA1c may be under‐utilised in cardiac centres with one study reporting only 43% while another UK‐based study reporting 12.5% of patients with ACS having had it performed.^20^, ^21^ Our service utilised HbA1c assessment in all patients admitted with ACS, identifying patients with new onset DM as well as aiding in the assessment of glycaemic control in those previously diagnosed with the condition. The increased frequency of the test performed and the greater number of newly diagnosed patients with DM identified highlights the feasibility and the clinical relevance of this approach in the screening and assessment of patients with DM when presenting with ACS. This, in turn, represents an opportunity to initiate glucose‐lowering medications with proven cardiovascular benefits such as SGLT2is and GLP1RA.^5^ Cardiovascular benefits with these classes of medications are seen irrespective of HbA1c levels and therefore, timely initiation can improve outcomes.^5^ The CDS led to a significant increase in overall and early initiation of SGLT2is, both at the time of discharge and within 12 months. One large recent multinational study showed that of patients admitted with NSTEMI and DM, only 9% received a prescription for SGLT2is at discharge^22^ while another Pan‐American^23^ study showed only 11.2% of patients with ischaemic heart disease and T2DM receiving the drug. Others report an even lower uptake at 1.8% in patients with an established cardiovascular event.^24^ While these statistics are similar to our findings before CDS initiation, a significant increase was noted after service commencement with prescriptions for the medicine class having more than doubled to 48.6% at discharge and 75.1% within 12 months by 2023. Similarly, the rate of GLP1RAs utilised in the above studies also remained low at 1.9%, 8.0% and 3.9%, respectively.^22^, ^23^, ^24^ Although the optimisation of GLP1RAs was included in the medicines optimisation protocol in the CDS, the recorded prescriptions did not differ significantly between the two groups in our analysis. This was due to the unavailability and worldwide shortage of these agents across the class in the later years of the study.^25^, ^26^ Nevertheless, patients in our analysis were still more frequently prescribed GLP1RAs (14%) in comparison to the above studies. Therefore, the CDS not only improved screening and monitoring but also aided in early optimisation of indicated therapies with proven cardiovascular benefit.

## 5 LIMITATIONS

Firstly, the above analysis represents registry data, and whilst every effort was made to ensure accurate data was collected and adjusted by clinicians specialising in cardiology and diabetes, this was only possible from a remote review of medical notes and discharge letters which may occasionally be limited in information. Similarly, some patients who moved out of the region had limited clinical post‐discharge information available to be added in the registry.

Secondly, the benefit in terms of reduction in mortality with the intervention in our analysis may be over‐estimated due to immortal bias favouring those that were included in the post‐intervention group during the implementation of phase 1 of the service (214/1073 (20%) with 128 pre‐intervention and 86 post‐intervention) whereby only those patients that were well enough to survive until the clinic appointment were included in the intervention arm. This may selectively bias patients who were well enough to be included in the post‐intervention arm whilst those that suffered a terminal event prior to the clinic were included in the pre‐intervention group. However, this number is small as only 40 patients from this time period had a terminal event. Of these, 8 were already included in the post‐intervention arm, thereby leaving 3.0% (32/1073) patients who were in the pre‐intervention arm and therefore subject to the bias. The rest of the patient allocation happened at the time of discharge and therefore not likely to have been affected with similar bias. This, therefore, represents a small proportion overall and less likely to account for the significant benefits seen above.

Finally, over the study 5‐year inclusion period, patients in the pre‐intervention group were from admissions of the earlier half whilst those in the post‐intervention were from a latter and more recent admission. A possible bias is that those admitted recently may have been subjected to better care and therefore improved outcomes unrelated to the CDS. However, key aspects of cardiovascular care and revascularisation practice including proportion of patients that received revascularisation and other key therapies aimed to help with cardiac remodelling and recovery including beta‐blockers, angiotensin and neprilysin inhibitors and mineralocorticoid receptor antagonists were similar (Tables 1 and 2). Other key patient characteristics such as baseline demographics (age, sex and smoking), clinical observations (blood pressure, heart rate, glucose and BMI), previous history of cardiovascular conditions, final diagnosis and left ventricular systolic function at discharge remained similar between the two groups. Therefore, despite the time difference, it is unlikely to have contributed significantly to the differences observed above.

Despite these limitations, this study shows promising results in the screening and optimisation of care of patients with DM presenting with ACS using a novel cardiodiabetes service comprising a specialist clinic and an in‐reach service. Further analysis using a greater number of patients in various clinical environments and hospital settings would further highlight the potential beneficial impact that may be seen with such a service.

## 6 CONCLUSION

The introduction of a joint‐speciality cardiodiabetes service, with active diabetes screening, management and medication optimisation, delivered by a multi‐professional team improved the care and survival of patients with DM admitted with AS in the above real‐world analysis.

## AUTHOR CONTRIBUTIONS

M.U.S. and K.L. conceived the idea, collected and analysed data and wrote the initial and reviewed the final draft. M.I. analysed data and reviewed and approved the final draft. B.S., A.R., C.E.H. and P.E.S. reviewed and approved the final draft. All authors are responsible for their contributions to the final draft and approved for submission.

## FUNDING INFORMATION

The Lincolnshire Heart Centre, United Lincolnshire hospitals NHS trust, received funding from Boehringer Ingelheim for the initial setup of the cardiodiabetes service led by K.L., B.S. and A.R.; however, no funding was allocated for the write‐up of this study and with no input in the above manuscript apart from the authors mentioned above.

## CONFLICT OF INTEREST STATEMENT

M.U.S. received travel and conference funding from Boehringer Ingelheim. None declared for any of the other authors.

## ETHICS STATEMENT

This study was a retrospective analysis of registry data for quality assurance purposes and was registered locally with the ULHT governance team as an audit and service improvement project (L0779). All data used in the analysis was pseudonymised.

## CONSENT

As this was a retrospective registry‐based analysis with pseudonymisation, additional consent from individual patients was not required and therefore not obtained. Retrospective analysis of pseudonymised data and therefore, consent for publication is not required.

## Supporting information


**Figure S1.** Variation in prescribing patterns for glucose‐lowering medications in patients with ACS at admission or follow‐up. Red Arrow highlights the initiation of Cardiodiabetic service in 2021. (Note: SGLT2is curve represents the proportion of patients amongst all patients included in the study including those with contraindications to SGLT2is therapy and prescribed at any time at admission or during follow‐up). Cardiodiabetic clinic started in summer 2021 and active in‐reach service commenced in autumn 2022.

**Figure S2.** Variations over 18 months for various blood test parameters. Blue = post‐intervention, Red = pre‐intervention.

**Appendix S1.** Standard operating procedures for the service attached as a Supporting Information.

## Data Availability

The data underlying this article will be shared on reasonable request to the corresponding author.
