# Supplementary figures and images for: Optimisation of care among patients with diabetes mellitus and acute coronary syndrome through a specialised cardiodiabetes service—A registry study
Source: Diabet Med. 2025 Apr 2;42(6):e70030. doi: 10.1111/dme.70030 (PMC12080982; doi:10.1111/dme.70030)

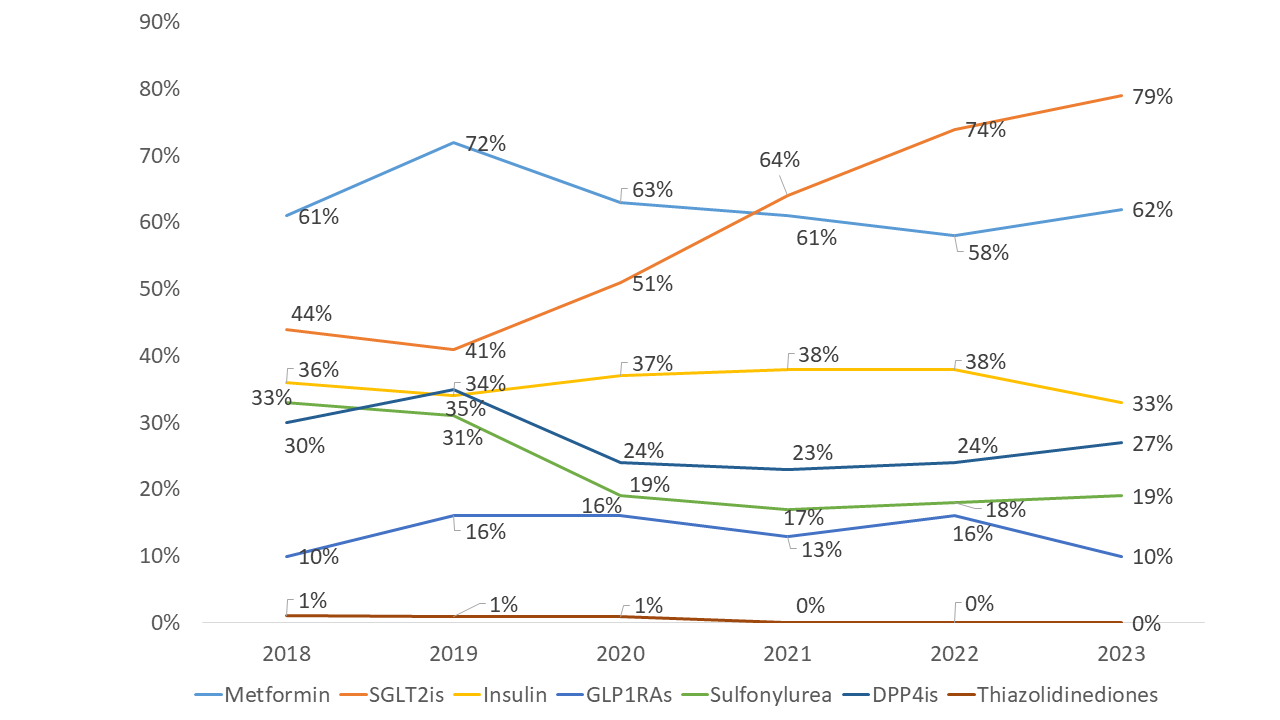

Supplement: Supplementary file 1 — Figure S1. Variation in prescribing patterns for glucose‐lowering medications in patients with ACS at admission or follow‐up. Red Arrow highlights the initiation of Cardiodiabetic service in 2021. (Note: SGLT2is curve represents the proportion of patients amongst all patients included in the study including those with contraindications to SGLT2is therapy and prescribed at any time at admission or during follow‐up). Cardiodiabetic clinic started in summer 2021 and active in‐reach service commenced in autumn 2022. [file DME-42-e70030-s001.tif]

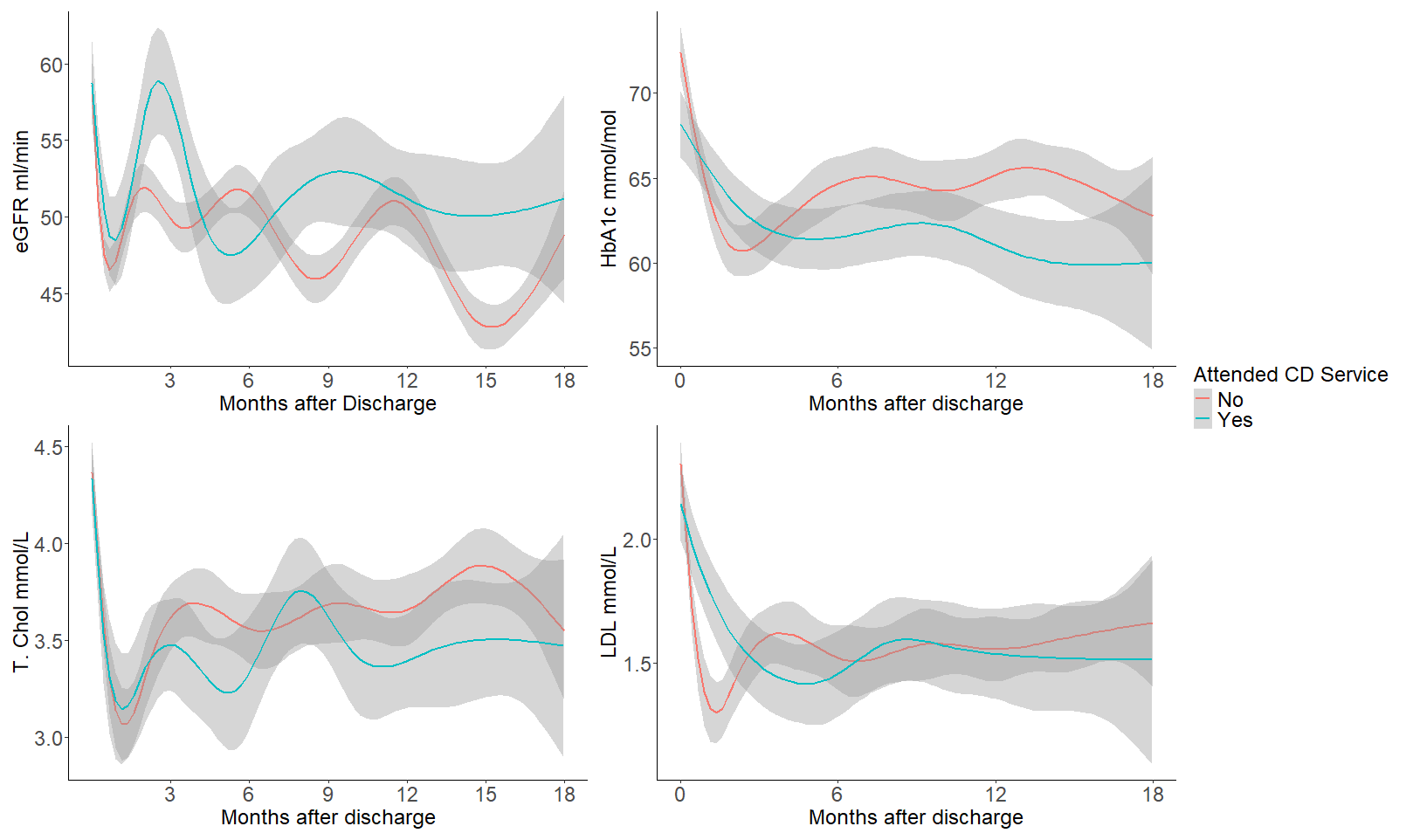

Supplement: Supplementary file 2 — Figure S2. Variations over 18 months for various blood test parameters. Blue = post‐intervention, Red = pre‐intervention. [file DME-42-e70030-s002.tiff]
