# Supplementary material for: Optimisation of care among patients with diabetes mellitus and acute coronary syndrome through a specialised cardiodiabetes service—A registry study
Source: Diabet Med. 2025 Apr 2;42(6):e70030. doi: 10.1111/dme.70030 (PMC12080982; doi:10.1111/dme.70030)
Supplement: Supplementary file 3 — Appendix S1. Standard operating procedures for the service attached as a Supporting Information. [file DME-42-e70030-s003.docx]

The Cardiodiabetic In-reach Programme

(For patients with Acute Coronary Syndrome and Diabetes)

Lincoln County Hospital

United Lincolnshire Hospitals NHS Trust
Lincoln

Table of Contents

[Background 3](#_Toc189305984)

[The cardiodiabetes service 4](#_Toc189305985)

[The cardio-diabetic multidisciplinary in-reach team 5](#_Toc189305986)

[Scope of patient care under the cardio-diabetic team 5](#_Toc189305987)

[Criteria for in-patient assessment and review 6](#_Toc189305988)

[Inclusion criteria 6](#_Toc189305989)

[Exclusion criteria 6](#_Toc189305990)

[Diabetic medication optimisation for patients with T2DM 7](#_Toc189305991)

[Metformin 7](#_Toc189305992)

[SGLT2is 7](#_Toc189305993)

[GLP1 RA 8](#_Toc189305994)

[Other agents 9](#_Toc189305995)

[Follow-up in the Cardio-Diabetes Out-Patient Clinic 9](#_Toc189305996)

[Management of hyperglycaemia in patients presenting with ACS 10](#_Toc189305997)

[Other domains of the cardiodiabetic in-reach programme: 15](#_Toc189305998)

[Improving in-patient screening investigations to identify patients with new type 2 DM – HbA1c screening 15](#_Toc189305999)

[Optimisation of diabetic and cardiovascular medications to improve outcomes 15](#_Toc189306000)

[Other Risk Factor optimisation for patients with T2DM: 16](#_Toc189306001)

[Special considerations for patients with a pre-diabetic range of HbA1c: 16](#_Toc189306002)

[Ensure timely out-patient follow-up in the Cardiodiabetic clinic for further optimisation and reduce the burden on other services 17](#_Toc189306003)

[Coordination with cardiodiabetic research programme for ongoing research studies 17](#_Toc189306004)

[The cardiodiabetes service registry database 18](#_Toc189306005)

[References 20](#_Toc189306006)

[Appendix 24](#_Toc189306007)

# Background

Diabetes remains a significant burden on healthcare systems and cardiovascular complications remain a leading cause of morbidity and mortality in these patients. There has been a rapid development in the management of patients with diabetes and ischaemic heart disease over the past few years, resulting in frequent updates in guidelines. Management of such patients can be complex and therefore, a multidisciplinary approach would potentially lead to better and more efficient patient management. A cardiodiabetic in-reach programme is a novel approach to the management of such patients while admitted to hospitals and then further assisting in appropriate outpatient follow-up.

# The cardiodiabetes service

The CDS is developed as a sub-service and an integral part of the Lincolnshire Heart Centre, United Lincolnshire Hospitals Trust (ULHT. The service was aimed for patients with known or newly diagnosed DM (type 1, type 2 and maturity-onset diabetes mellitus (MODY)) presenting with an ACS (inclusive of ST-elevation myocardial infarction [STEMI], non-ST elevation myocardial infarction [NSTEMI] or troponin negative unstable angina) ^1–3^ and admitted to ULHT hospitals (i.e., Lincoln County Hospital, Grantham District Hospital and Pilgrim Boston Hospital). Patients who are pre-diabetic or non-diabetic, or, had a primary diagnosis other than ACS such as type 2 myocardial infarction or arrhythmias, are not eligible for the service. The service is delivered by a multi-professional team of cardiology and diabetes consultant physicians, physician fellows and advanced clinical practitioners.

## History

The CDS was developed in two phases. The first phase started in summer 2021 and involved setting up of the cardiodiabetes clinic; a telephone clinic run by cardiology advanced clinical practitioners where patients were called and reviewed virtually by the cardiodiabetic team. A multi-disciplinary team meeting comprising cardiology and diabetes consultants, clinical fellows and advanced clinical practitioners was held prior to each clinic. All patients booked into the cardiodiabetes clinic were discussed in the multi-disciplinary team meeting with a holistic clinical approach in optimisation of management with a focus on the cardiovascular, diabetes and lipid risk factor profiles. This included ensuring timely initiation of glucose-lowering medicines with proven cardiovascular benefits, repeating HbA1c and lipid profiles to assess adequate control and commencing guideline-directed adjuvant lipid-lowering therapies where indicated and previously agreed in the standard operating procedures (appendix 1). Following the telephone clinic review, patients were discharged back to the care of their local primary care service with an ongoing cardiodiabetes management plan ^3^. Patients who required further cardiology or diabetes follow-up or intervention were referred to the respective service appropriately as determined by Lincolnshire commissioning pathways.

The second phase, implemented since autumn 2022, involved the initiation of an active DM screening programme in all patients admitted with ACS at ULHT hospitals. Individuals who met the clinical criteria for DM ^4^ or those with previously established DM, were then further reviewed and medications optimised as allowed by their clinical condition whilst still admitted in the hospital. These patients were then directly booked in for a review in the Post-ACS CDS initiated in phase 1, within 3 months following discharge, for further optimisation in a staged manner^2^.

# The cardio-diabetic multidisciplinary in-reach team

The cardio-diabetic in-reach programme is be led by a clinical research fellow, with supervision from Cardiology and Diabetes specialist consultants and assisted by the cardiology advanced nurse practitioners and diabetic specialist nurses and comprises thrice weekly ward rounds and discussion in the cardio-diabetic MDT once weekly as needed. Patient management are optimised and then appropriate follow-up arranged as an outpatient.

## Scope of patient care under the cardio-diabetic team

The cardiology department at Lincoln County Hospital comprises CCU, Johnson and Cardiac short-stay wards, where patients presenting with various cardiovascular conditions are managed. Acutely unwell patients, including ST elevation myocardial infarction (STEMI), pulmonary oedema and shock are managed in CCU, which is the high dependency unit, and once stable, they are then stepped down to Johnson and short-stay wards. Other conditions managed in the latter wards include Non- non-ST-elevation myocardial infarction (NSTEMI), unstable angina, heart failure, arrhythmias and endocarditis. The team caring for these patients includes Consultants, assisted by Junior and middle-grade doctors and Advanced Care Practitioners (ACPs).

The cardio-diabetic in-reach team screens and review patients in these wards who meet the criteria using information gained from a combination of electronic and team handovers. Patients that meet the criteria (to check HbA1c with ACS if not performed) are then be approached. Patients’ medications are reviewed and amended as appropriate.

## Criteria for in-patient assessment and review

### Inclusion criteria

- Known or newly diagnosed Diabetes mellitus (to Check HbA1c if not done)
- Patients presenting with chest pain and diagnosed with acute myocardial infarction (STEMI/NSTEMI) or Unstable angina
- Haemodynamically stable (if not, delay the review until after haemodynamic stability of more than 24hrs)

### Exclusion criteria

- Type 2 MI / Myocardial injury
- Acutely Unwell patient
- cardiogenic shock
- Acute pulmonary oedema
- Other causes of chest pain e.g. Aortic Stenosis, or non-cardiac causes such as pulmonary embolism.

## Diabetic medication optimisation for patients with T2DM

A target HbA1c of ≤ 53 mmol/L should be sought in the management of these patients for long-term reduction in cardiovascular outcomes^5^. Whilst some groups of medications attempt to reduce the HbA1c levels, and therefore may not be indicated if adequate control is achieved, others (e.g. SGLT2is and GLP1-RA) confer additional benefits in reducing cardiovascular mortality, overall mortality and further cardiac events, irrespective of the HbA1c levels and should therefore be considered if not contraindicated otherwise (Figure1).

### Metformin

Metformin is known to reduce cardiovascular and all-cause mortality in patients with T2DM^6^. Therefore, this should be commenced if not already on this, unless contraindicated, HbA1c <48 mmol/mol or GFR <45 ml/min. The starting dose should be 500 mg twice daily which may be up titrated as needed.

### SGLT2is

These medications are now known to reduce cardiovascular outcomes in patients with T2DM and atherosclerotic cardiovascular disease (ASCVD)^7–10^. Therefore, these should also be considered the first line in addition to Metformin. Empagliflozin or Dapagliflozin at 10 mg once daily to be commenced if no contraindications which include allergic reactions, low systemic blood pressure (<100 systolic), acute kidney injury, end-stage renal disease (GFR <15 for Dapagliflozin and <20 for Empagliflozin), active diabetic foot disease or limb ischaemia, and previous history of diabetic ketoacidosis (DKA).

These medicines should not be used in those with Type 1 Diabetes Mellitus due to the risk of (Euglycemic) diabetic ketoacidosis. Patients should also be given information leaflets on sick day rules when commencing on SGLT2i. Concurrent medication note: If a patient has previously been commenced on Gliclazide, then the Gliclazide may be continued at the usual dose if the HbA1c is >64mmol/mol, or reduced to half the dose if HbA1c is 54-64 mmol/mol and stopped entirely if <54 mmol/mol. Similarly, if on insulin, then continue routine dose if HbA1c ≥ 64 mmol/mol or half dose if <64 mmol/mol. The patient would need to be educated on home ketone monitoring and symptoms of DKA (Euglycemic). Patients on Insulin will continue with regular BM monitoring.

### GLP1 RA

These are now shown to also offer a significant reduction in cardiovascular outcomes in patients with ASCVD and T2DM and are recommended for use in these individuals^11^. Semaglutide is available in subcutaneous (0.25mg injection, once a week) and oral forms (3 mg once daily). The dose is up-titrated over the following 8 weeks. It is recommended for use in obese patients with BMI ≥35 and BMI ≥30 where weight loss is desirable for health reasons. Dose reduction for gliclazide and insulin is the same as for SGLT2is described above. Semaglutide is relatively contraindicated in those with proliferative and preproliferative retinopathy. Please contact the diabetes Team for alternatives

### Other agents

If an acceptable control of HbA1c is not achieved, then additional medications may be introduced including DPP-4i (Linagliptin, 5mg once daily), thiazolidinediones e.g. Pioglitazone 15-30 mg once daily (to be avoided in heart failure), sulfonylureas (e.g. Gliclazide 40-80 mg once daily) or basal insulin^11^.

# Follow-up in the Cardio-Diabetes Out-Patient Clinic

Patients with known or newly diagnosed T2DM and T1DM will have outpatient follow-up arranged for the Cardio-Diabetes Clinic in 3 months. This will be noted by:

- EDD, updated with “Cardiodiabetes” outpatient follow-up (Discharge team)
- Patient details sent to the administration team to ensure that the above follow-up is arranged (Cardiodiabetes in-reach team)

In addition, the EDD plan should mention a request to the GP surgery to repeat the lipid profile and HbA1c at 10 weeks following discharge.

# Management of hyperglycaemia in patients presenting with ACS

It is known that patients with T2DM and ACS have higher long-term morbidity and mortality. Hyperglycaemia on admission is an even stronger predictor of poorer outcomes, irrespective of a previous diagnosis of diabetes mellitus^12,13^. In addition, Kosiborod *et. al.* demonstrated that normalisation of this admission hyperglycaemia (≤6mmol/L), resulted in reduced mortality in these patients^14^. The effects of insulin infusion treatment as a strategy to reduce blood glucose levels have conflicting evidence. The first Diabetes Mellitus, Insulin Glucose Infusion in Acute Myocardial Infarction (DIGAMI) trial showed longer survival in patients with hyperglycaemia and ACS treated with insulin^15^. However, the DIGAMI 2 trial did not show any obvious benefit with this treatment, although the glycaemic control between the interventional and control arms did not differ much and may represent a reason for the lack of difference in outcomes^16^. Kosiborod *et.al.* showed that the beneficial effects seen with normoglycaemia after ACS were seen in patients irrespective of whether or not this was achieved with the help of insulin^14^. However, ACS does represent a stress condition with resulting hyperglycaemia which may not be well controlled with oral medications in the acute phase. Therefore, a variable rate insulin infusion (VRIII) may be a preferred option to achieve this control.

In contrast, severe hypoglycaemia (BM ≤3.0) in the context of ACS is known to result in increased mortality and should be avoided^17^. Previous trials showed an incidence of 10 to 15% of patients experiencing hypoglycaemia when treated with Insulin. To assess this further, the UK-based, multi-centre “Evaluation of the safety and efficacy of a variable rate intravenous insulin infusion in the management of hyperglycaemia in acute coronary syndrome” (TITAN-ACS) study was performed. Of the total of 776 patients assessed, 32 (4.1%) patients were recorded to have severe hypoglycaemia, with the majority having a single episode. This was more common in the early period of the infusion (initial 5 hours), for those whose capillary blood glucose was between 4 to 8 mmol/L and whose body weight was less than 74kg^18^. However, no increased mortality^18^ at 30 days was noted and the regimen was considered safe and effective, although with rate adjustments required at extremes of weights. In the “Intensive versus conventional glucose control in critically Ill patients,” (NICE-SUGAR) study, intensive glucose control (4.5 to 6.0 mmol/L) was compared with conventional glucose control (≤10.0 mmol/L) in 6104 patients requiring at least 3 days of intensive care treatment. Although this was not a purely ACS study, patients in the former group had a significantly higher mortality. Therefore, based on the above discussion, and as per NICE recommendations, we have produced the following guidance on patients admitted with confirmed or suspected ACS and blood capillary glucose measurements confirming hyperglycaemia (≥10 mmol/L)^19^.

| Variable Rate IV Insulin Infusion CCU:  1. Print off protocol  2. Attach Patient ID Label  3. Staple to IV Insulin Prescription Chart | |  | Patient ID Label  Name  DOB  NHS number | |  |
| --- | --- | --- | --- | --- | --- |
|  | |  |  |  |  |
| **INSULIN / DEXTROSE INFUSION PROTOCOL FOR ACUTE CORONARY SYNDROME**  **(ACS) PATIENTS WITH HYPERGLYCAEMlA**^18,20^ | | | | |  |
| **Criteria for use in patients on CCU, during first 24 hours of admission:**   - Patients with ACS where the probability of an elevated troponin is high i.e., dynamic ECG changes **AND** random capillary blood glucose (CBG) ≥10mmol/L (taken as soon as possible) - Confirm Troponin T+ve (do not wait for Troponin T before starting infusion) | | | | |  |
| **Exclusion criteria:**   - Severe non-cardiac co-morbidity with an estimated prognosis of < 6 months - Complex metabolic disorders likely to have an impact on glycometabolic control - Pregnancy - Patients in whom transfer to another hospital is imminent | | | | |  |
| **Investigations on admission:**   - Laboratory glucose, renal function tests (For admission K^+^ and then repeat at 24 hours to ensure in satisfactory range), liver function tests & lipid profile - HbA1c - IMPORTANT - for diagnostic purposes (does not need to be fasting) | | | | |  |
| **Existing diabetes treatment (If any):**   - Stop existing oral diabetes therapy or s/c insulin for the duration of insulin infusion, except long-acting (basal) insulin (e.g. Lantus or Levemir or Tresiba (Degludec) or Insulatard or Insuman Basal or Humulin I or Porcine/Bovine Isophane) **which should be continued during the IV insulin infusion**. | | | | |  |
| **SETTING UP THE INFUSION**   - Commence syringe driver with 50 units of soluble insulin in 50ml of 0.9% sodium chloride. Adjust insulin infusion according to hourly CBG reading (see chart below). - If the surgical patient then commences a separate infusion of 500ml premixed glucose 5%/ NaCl 0.45%/ KCL 0.15% (10 mmol) set rate at 125 ml/hr. For non-surgical patients, use 5% w/v Glucose with 20 mmol potassium chloride (0.15% w/v) in 1L at the recommended rate of 83 mls/hr. Consider a slower rate in patients with heart failure. - If concern fluid overload/pulmonary oedema use 500 ml Glucose 10% with KCL 10mmol at 15ml/hr - If potassium >5.0mmol/L, eGFR<15ml/min/1.73m2 or on Dialysis, then consider Glucose 10% 500 ml bag at 50ml/hr | | | | |  |
| **Monitoring and Insulin adjustment:** **Aim Capillary Blood Glucose (CBG) 5 to 10mmoI/L** | | | | |  |
| Glucose | Insulin Infusion Rate ml/ hour | | | Practical points   - Check CBG hourly until within range (5-10 mmol/L) for two consecutive hours, then reduce CBG checks to 2 hourly. - If CBG is Within the range of 5-10mmol/L for 6 hours, the Infusion may be withdrawn. Record the date and time that the Infusion is stopped on the Insulin Infusion chart. - If CBG is in range and Infusion is stopped, recheck CBG at 1 and 2 hours and If CBG rises to > 10mmot/L, restart Infusion and continue as before to 24hr point.   Refer patients to Inpatient DSN |  |
| ≤4mmol/L   | 0.5ml (if Long-acting background insulin has been continued, then stop infusion and record date/time.  Recheck CBG at 1 and 2 hrs. Restart infusion if CBG >10mmol/L at 2 hours).  Treat hypoglycaemia as per Trust Guidelines. | | |  |  |
| 4-1 — 7.0 mmol/L | 1ml (=1 unit/hour) | | |  |  |
| 7.1—9.0 mmol/L | 2ml | | |  |  |
| 9.1 —11 mmol/L | 3ml | | |  |  |
| 11.1—14.0mmol/L | 4ml | | |  |  |
| 14.1—17.0mmol/L | 5ml | | |  |  |
| ≥17.0-20.0 mmol/L | 6 ml. If CBG >20 mmol/L, please seek diabetes or medical team advice | | |  |  |
| 24 hour point: If patient eating/drinking stop infusions (record date/time), if appropriate return to usual diabetes medication (plan ahead - ask medics to prescribe in advance). If on short/intermediate acting insulin ensure overlap of 30 minutes (will need to take place when patient usually administers insulin, I.e. meal time.) Patients with known diabetes: check CBG levels pre-meal and bed during admission. | | | | |  |
|  |  |  |  |  |  |
|  |  |  |  |  |  |


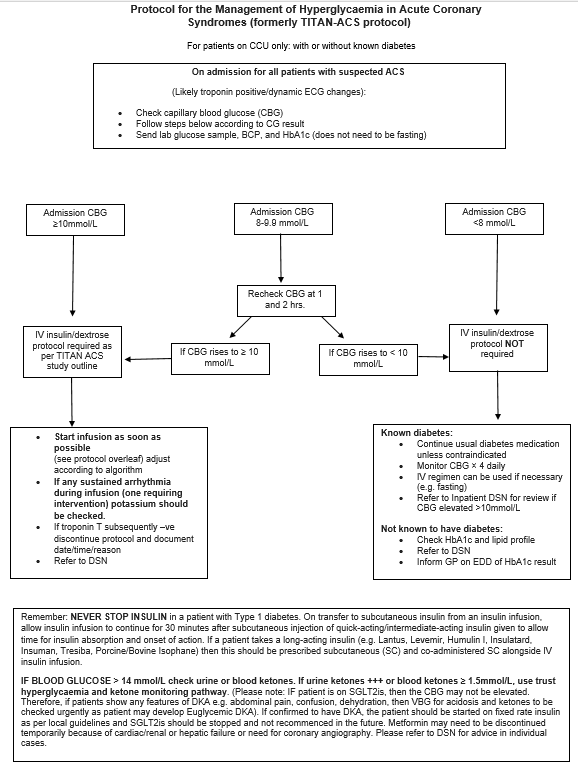


# Other domains of the cardiodiabetic in-reach programme:

## Improving in-patient screening investigations to identify patients with new type 2 DM – HbA1c screening

Recent department audit has shown that there are patients admitted with ACS that are not having HbA1c measured. Therefore, an opportunity is lost to identify patients with undiagnosed new T2DM, as well as the assessment of glycaemic control of known T2DM patients. These patients (newly undiagnosed T2DM and uncontrolled known T2DM) are at higher risk of further cardiovascular events. Guidelines now recommend the use of additional medical therapy, including SGLT2i, to further reduce the risk of such events^11^. The cardiodiabetic in reach programme will review such patients and request HbA1c, if not already done so and identify such high risk patients, enabling inpatient review, optimisation and ensuring appropriate follow up.

## Optimisation of diabetic and cardiovascular medications to improve outcomes

Multiple randomised control trials have shown the benefit of SGLT2i, GLP1-RA and metformin in patients with T2DM and AMI. The in-reach team will review the notes and medications of these patients and update the management plan as per locally developed guidelines (Figure 1).

## Other Risk Factor optimisation for patients with T2DM:

In addition to managing diabetes, other risk factors need to be addressed and treatment optimised. If not contraindicated, patients should be commenced on ACEi/ARBs. Systemic blood pressure should be well controlled and ideally <140/90 mmHg, although reduction to 120/80 mmHg has shown to further reduce cardiovascular outcomes, although with an increase in adverse events from intensive anti-hypertensive therapy^21,22^. Lipid-lowering treatment in the form of high-dose atorvastatin (80mg) should be commenced with the aim of lowering LDL to <1.4 mmol/L. If not achieved with statins alone then add-on therapy in the form of ezetimibe, inclisiran (LDL >2.6 mmol/L) and PCSK9is (LDL> 3.5 mmol/L) should be considered^23–25^. Additionally, a fasting Triglycerides level of <1.7 mmol/L should be aimed for and to consider Icosapent ethyl add-on therapy if elevated and LDL levels are less than 2.6 mmol/L^26^. In addition to medical therapy, patients should be counselled on diet and lifestyle changes, aiming for total fat intake of <30% of total energy intake, saturated fats <7% and increasing physical activity to >150 minutes of moderate or >75 minutes of vigorous activity per week^27^. Weight should be reduced aiming for a BMI of <25 kg/m^228^. Further advice on low-risk alcohol intake and smoking cessation should also be provided and reinforced^29,30^.

## Special considerations for patients with a pre-diabetic range of HbA1c:

Patients with HbA1c 42-47 mmol/mol are classified as pre-diabetic unless previously confirmed to have diabetes. Whilst prediabetic patients may be at slightly less risk of cardiovascular outcomes in comparison to diabetic patients, a significant proportion of pre-diabetic patients may progress to develop diabetes. Therefore, these patients need to be referred to diabetes preservation services by the GP and this must be highlighted in the EDD discharge plan. Care must be taken as some diabetic patients have HbA1c in the prediabetic (or even normal) range. However, they remain diabetic and should be followed up in the cardiodiabetic clinic.

## Ensure timely out-patient follow-up in the Cardiodiabetic clinic for further optimisation and reduce the burden on other services

A local audit of the cardio-diabetes clinic showed that only 33% of Diabetic patients were being followed in the cardiodiabetic clinic. Of these, only 43% were being seen within 12 weeks. With active patient reviews by the cardiodiabetic team, potential dates for follow-up will be identified for the administration team to book the patient in, therefore, ensuring as timely follow-up as appropriate.

## Coordination with cardiodiabetic research programme for ongoing research studies

Lincoln County Hospital has an active, and rapidly growing, Cardiovascular Research Team. In collaboration with the University of Lincoln, the research team is carrying out a mechanistic study to assess the underlying mechanism of beneficial effects of SGLT2i in diabetic patients with AMI. In addition, the study will also highlight the potential benefits of early versus delayed start of SGLT2i in these patients. The results of this study would further point towards possible additional treatment pathways that might help symptoms and prognosis in these conditions. To this effect, the team has secured a grant from the European Foundation for the Study of Diabetes which further underscores the importance of high levels of research in this relatively research free zone.

Similarly, the team are also leading the Cardiodiabetic Early Intervention Study (CAMIS) which is a retrospective-prospective registry-based observational study to further assess the benefit of early commencement of SGLT2i in patients with AMI and T2DM on hospitalisation for heart failure, further MIs and death.

The Cardiodiabetic in-Reach Programme will be invaluable in the screening and recruitment of participants to both these studies, as well as other studies run in parallel by the research and development department.

## The cardiodiabetes service registry database

A registry was set up for the operational elements of the service and audit. All patients with an ACS admitted to ULHT were recorded in the local Myocardial Infarction National Audit Project registry from where eligible patients were identified, along with those identified by the inpatient DM screening programme initiated as part of phase 2, and included in the cardiodiabetic registry. Patient clinical data were collected from the shared pan-Lincolnshire primary and secondary patient record CarePortal system and included clinical observations, co-morbid conditions such as established ischaemic heart disease, heart failure, chronic kidney disease (CKD) and con-current medication use at baseline ^31^. All subsequent urgent or emergent unplanned admissions were then identified using the same system and relevant details, including primary diagnosis and outcomes, recorded in the database. A dedicated pan-Lincolnshire information technology program, the WebV system, was used to collect blood test results comprising lipid profile, renal function tests and HbA1c, both at baseline and follow up^32^. Data was recorded until date of death or last recorded visit noted in the hospital or general practitioner records, the latter taken as censor date.

# References

1. Ingelheim B. United Lincolnshire Hosps NHS Trust | Cardiometabolic Care. Boehringer Ingelheim. August 9, 2022. Accessed August 16, 2024. https://www.boehringer-ingelheim.com/uk/partnering/human-health-partnering/partnering-interests/collaborative-working/united-lincolnshire

2. Platform FC. FutureNHS Case Study Hub : (Part 2) Establishing a novel Cardio-Diabetic pathway, optimising patient care, improving cardiovascular and diabetes outcomes in diabetic patients with acute coronary syndrome : The Cardio-Diabetic in-reach programme. Accessed August 16, 2024. https://future.nhs.uk/about/

3. Platform FC. FutureNHS Case Study Hub : (Part 1) Establishing a novel cardio-metabolic pathway, optimising patient care, improving cardiovascular and diabetes outcomes in diabetic patients with acute coronary syndrome. Accessed August 16, 2024. https://future.nhs.uk/about/

4. Marx N, Federici M, Schütt K, et al. 2023 ESC Guidelines for the management of cardiovascular disease in patients with diabetes: Developed by the task force on the management of cardiovascular disease in patients with diabetes of the European Society of Cardiology (ESC). *Eur Heart J*. Published online August 25, 2023:ehad192. doi:10.1093/eurheartj/ehad192

5. NICE. Type 2 diabetes in adults: management. Published online 2022.

6. Han Y, Xie H, Liu Y, Gao P, Yang X, Shen Z. Effect of metformin on all-cause and cardiovascular mortality in patients with coronary artery diseases: a systematic review and an updated meta-analysis. *Cardiovasc Diabetol*. 2019;18(1):96. doi:10.1186/s12933-019-0900-7

7. Neal B, Perkovic V, Mahaffey KW, et al. Canagliflozin and Cardiovascular and Renal Events in Type 2 Diabetes. *N Engl J Med*. 2017;377(7):644-657. doi:10.1056/NEJMoa1611925

8. Wiviott SD, Raz I, Bonaca MP, et al. Dapagliflozin and Cardiovascular Outcomes in Type 2 Diabetes. *N Engl J Med*. 2019;380(4):347-357. doi:10.1056/NEJMoa1812389

9. Zinman B, Wanner C, Lachin JM, et al. Empagliflozin, Cardiovascular Outcomes, and Mortality in Type 2 Diabetes. *N Engl J Med*. 2015;373(22):2117-2128. doi:10.1056/NEJMoa1504720

10. Zelniker TA, Wiviott SD, Raz I, et al. SGLT2 inhibitors for primary and secondary prevention of cardiovascular and renal outcomes in type 2 diabetes: a systematic review and meta-analysis of cardiovascular outcome trials. *The Lancet*. 2019;393(10166):31-39. doi:10.1016/S0140-6736(18)32590-X

11. Cosentino F, Grant P, Aboyans V, Bailey C. 2019 ESC Guidelines on diabetes, pre-diabetes, and cardiovascular diseases developed in collaboration with the EASD: The Task Force for diabetes, pre-diabetes, and cardiovascular diseases of the European Society of Cardiology (ESC) and the European Association for the Study of Diabetes (EASD). *Eur Heart J*. 2020;73(5):404. doi:10.1016/j.rec.2020.04.007

12. Gholap NN, Mehta RL, Ng L, Davies MJ, Khunti K, Squire IB. Is admission blood glucose concentration a more powerful predictor of mortality after myocardial infarction than diabetes diagnosis? A retrospective cohort study. *BMJ Open*. 2012;2(5):e001596. doi:10.1136/bmjopen-2012-001596

13. Nicolau JC, Serrano CV, Giraldez RR, et al. In patients with acute myocardial infarction, the impact of hyperglycemia as a risk factor for mortality is not homogeneous across age-groups. *Diabetes Care*. 2012;35(1):150-152. doi:10.2337/dc11-1170

14. Kosiborod M, Lam CSP, Kohsaka S, et al. Cardiovascular Events Associated With SGLT-2 Inhibitors Versus Other Glucose-Lowering Drugs: The CVD-REAL 2 Study. *J Am Coll Cardiol*. 2018;71(23):2628-2639. doi:10.1016/j.jacc.2018.03.009

15. Malmberg K, Rydén L, Efendic S, et al. Randomized trial of insulin-glucose infusion followed by subcutaneous insulin treatment in diabetic patients with acute myocardial infarction (DIGAMI study): effects on mortality at 1 year. *J Am Coll Cardiol*. 1995;26(1):57-65. doi:10.1016/0735-1097(95)00126-k

16. Malmberg K, Rydén L, Wedel H, et al. Intense metabolic control by means of insulin in patients with diabetes mellitus and acute myocardial infarction (DIGAMI 2): effects on mortality and morbidity. *Eur Heart J*. 2005;26(7):650-661. doi:10.1093/eurheartj/ehi199

17. Garg R, Hurwitz S, Turchin A, Trivedi A. Hypoglycemia, with or without insulin therapy, is associated with increased mortality among hospitalized patients. *Diabetes Care*. 2013;36(5):1107-1110. doi:10.2337/dc12-1296

18. Hammersley MS, Rayman G, Winocour P, Weston C, Birkhead J, Group OBOTTAS. An evaluation of the safety and efficacy of a variable rate intravenous insulin infusion in the management of hyperglycaemia in acute coronary syndrome: experience of the TITAN-ACS. *Br J Diabetes*. 2015;15(4):173-179. doi:10.15277/bjdvd.2015.041

19. Corbett SJ. NICE recommendations for the management of hyperglycaemia in acute coronary syndrome. *Heart*. 2012;98(16):1189-1191. doi:10.1136/heartjnl-2012-302421

20. NICE-SUGAR Study Investigators, Finfer S, Chittock DR, et al. Intensive versus conventional glucose control in critically ill patients. *N Engl J Med*. 2009;360(13):1283-1297. doi:10.1056/NEJMoa0810625

21. Recommendations | Hypertension in adults: diagnosis and management | Guidance | NICE. Accessed June 17, 2020. https://www.nice.org.uk/guidance/ng136/chapter/Recommendations

22. The SPRINT Research Group. A Randomized Trial of Intensive versus Standard Blood-Pressure Control. *N Engl J Med*. 2015;373(22):2103-2116. doi:10.1056/NEJMoa1511939

23. 1 Recommendations | Ezetimibe for treating primary heterozygous-familial and non-familial hypercholesterolaemia | Guidance | NICE. February 24, 2016. Accessed April 10, 2023. https://www.nice.org.uk/guidance/ta385/chapter/1-Recommendations

24. 1 Recommendations | Inclisiran for treating primary hypercholesterolaemia or mixed dyslipidaemia | Guidance | NICE. October 6, 2021. Accessed April 10, 2023. https://www.nice.org.uk/guidance/ta733/chapter/1-Recommendations

25. 1 Recommendations | Alirocumab for treating primary hypercholesterolaemia and mixed dyslipidaemia | Guidance | NICE. June 22, 2016. Accessed April 10, 2023. https://www.nice.org.uk/guidance/ta393/chapter/1-Recommendations

26. Overview | Icosapent ethyl with statin therapy for reducing the risk of cardiovascular events in people with raised triglycerides | Guidance | NICE. July 13, 2022. Accessed April 10, 2023. https://www.nice.org.uk/guidance/ta805

27. 1 Recommendations | Cardiovascular disease: risk assessment and reduction, including lipid modification | Guidance | NICE. Accessed April 30, 2020. https://www.nice.org.uk/guidance/cg181/chapter/1-Recommendations

28. Recommendations | Obesity: identification, assessment and management | Guidance | NICE. November 27, 2014. Accessed April 10, 2023. https://www.nice.org.uk/guidance/cg189/chapter/Recommendations

29. Alcohol consumption: advice on low risk drinking. GOV.UK. Accessed April 10, 2023. https://www.gov.uk/government/publications/alcohol-consumption-advice-on-low-risk-drinking

30. Overview | Tobacco: preventing uptake, promoting quitting and treating dependence | Guidance | NICE. November 30, 2021. Accessed April 10, 2023. https://www.nice.org.uk/guidance/ng209

31. Lincolnshire Partnership NHS Foundation Trust. Lincolnshire’s new Care Portal :: Lincolnshire Partnership NHS Trust. Accessed September 21, 2024. https://www.lpft.nhs.uk/news-and-events/news/lincolnshires-new-care-portal

32. Systems W. WebV Systems. WebV Systems. Accessed September 21, 2024. https://webv-solutions.com/

# Appendix


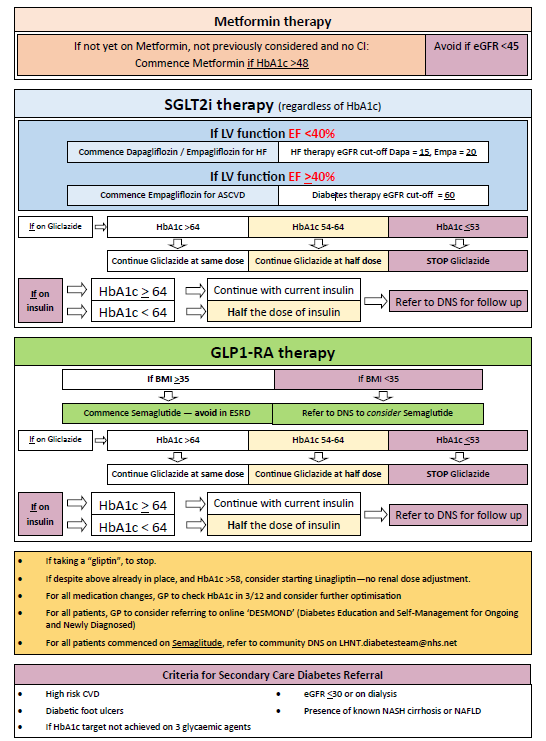


Figure 1: Local guidelines for medication optimisation of patients with T2DM and ACS.
